# Low heart-type fatty acid binding protein level during aging may protect down syndrome people against atherosclerosis

**DOI:** 10.1186/1742-4933-10-2

**Published:** 2013-01-22

**Authors:** Elena Vianello, Giada Dogliotti, Elena Dozio, Massimiliano Marco Corsi Romanelli

**Affiliations:** 1 Dipartimento di Scienze Biomediche per la Salute, Cattedra di Patologia Clinica, Università degli Studi di Milano, Via Mangiagalli 31, Milan, 20133, Italy; 2 U.O.C. di Patologia Clinica, Dipartimento dei Servizi Sanitari di Diagnosi e Cura – Medicina di Laboratorio, IRCCS Policlinico San Donato, Piazza E. Malan, 20097 San Donato Milanese, Milan, Italy

**Keywords:** Aging, Atherosclerosis, Down syndrome, Heart-type fatty acid binding protein

## Abstract

**Background:**

Aging is considered an important independent risk factor for atherosclerosis. Down syndrome people (DS) display an accelerated aging process compared to healthy subjects, anyway they are relatively resistant to developing atherosclerosis. The mechanisms involved in such protective effect are not well known. Since heart-type fatty acid binding protein (H-FABP) is a protein involved in the transport of fatty acids and it has been recently correlated with the presence of atherosclerosis, we aimed to measure H-FABP level both in DS and in healthy subjects during aging to evaluate the association between this molecule, aging and atherosclerosis.

**Findings:**

We quantified plasmatic H-FABP level in three groups of male DS and age-matched healthy subjects (children, age 2–14 years; adults, age 20–50 years; elderly, > 60 years) using a biochip array analyzer. We observed that aging is associated with increased H-FABP level in healthy subjects but not in DS which display both the same protein level in the different ages of life and have also lower level compared to their age-matched healthy subjects.

**Conclusion:**

Reduced H-FABP level during aging in DS may play a protective role against atherosclerosis. The potential involvement of H-FABP in the relationship between aging, atherosclerosis and development of coronary artery disease needs further investigations.

## Introduction

Atherosclerosis is a chronic inflammatory response of the walls of arteries mainly due to a deregulated lipid metabolism and promoted by the local accumulation of macrophages [1]. In addition to the well known risk factors, including hypertension, diabetes, low-density lipoprotein cholesterol and smoking, increasing evidence suggested that aging is also an important independent risk factor for atherosclerosis, persisting also when other known factors are controlled [2].

Heart-type fatty acid binding protein (H-FABP) is a small intracellular protein involved in the transport of hydrophobic long-chain fatty acids from the cell membrane inside the cells. In addition, it also promotes the expression of different pro-inflammatory cytokines and is a powerful regulator of the mitochondrial beta-oxidative system in the heart [3,4]. A recent study indicated that circulating H-FABP is positive correlated with intima-media thickness and may represent a new possible diagnostic biomarker for early atherosclerosis [5].

Several studies shown that people affected by down syndrome (DS) are relatively resistant to developing atherosclerosis and coronary artery diseases despite the presence of an unfavourable plasma lipid profile and are generally considered an “atheroma-free” model [6-8].

For these reasons, due to the absence of atherosclerosis in DS, in the present study we aimed to measure H-FABP level both in DS and in healthy subjects during aging to evaluate the association between this molecule, aging and atherosclerosis.

### Subjects and methods

#### Subjects

Three groups of male DS and age-matched healthy subjects were studied: the first consisted of 23 DS and 20 healthy children (age 2–14 years); the second of 14 DS and 20 healthy adults (age 20–50 years); the third group of 13 DS and 20 healthy elderly (> 60 years). All DS were assessed by clinical examination and karyotype analysis; they had mild and variable degree of mental retardation, no other pathological conditions at the time of the study and were in good health. The project was approved by University of Milan Ethics Committee and by the Fondazione Antoniana of Bologna, Italy.

#### FABP assay

Plasma was obtained by centrifugation at 1500 g for 15 min, transferred into coded plastic tubes, rapidly frozen and stored at −20°C until analysis. H-FABP was quantified using a biochip array analyzer (Evidence Invatigator, Randox Ltd., Crumlin, UK). A biochip is a solid substrate supporting an array of discrete test regions, with immobilized antigen-specific antibodies. After an immuno-enzymatic reaction, each spot generates a chemiluminescent signal on the array which is captured by a charge-coupled camera (CCD-camera) and converted by an image processing software to provide results comparable with calibration curves.

#### Statistical analysis

The results are given as mean ± standard deviation (SD). Comparison between groups was performed using Student’s two-tailed *t* test. A p value < 0.05 was considered significant. Analyses were performed using GraphPad Software (San Diego, CA).

## Results

**Figure 1:**
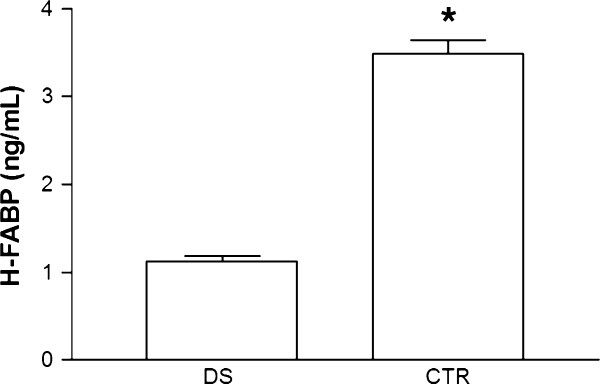
**H-FABP level in down syndrome people (DS) compared to healthy subjects (CTR).** H-FABP level was evaluated in DS and in CTR groups (age range 2–65 years). *p < 0.0001 *vs.* CTR.

By comparing DS and healthy subjects groups (age range 2–65 years) we observed lower H-FABP level in DS group (1.13 ± 0.34 ng/mL *vs.* 3.49 ± 0.96 ng/mL; p < 0.0001) (Figure 1).

**Figure 2:**
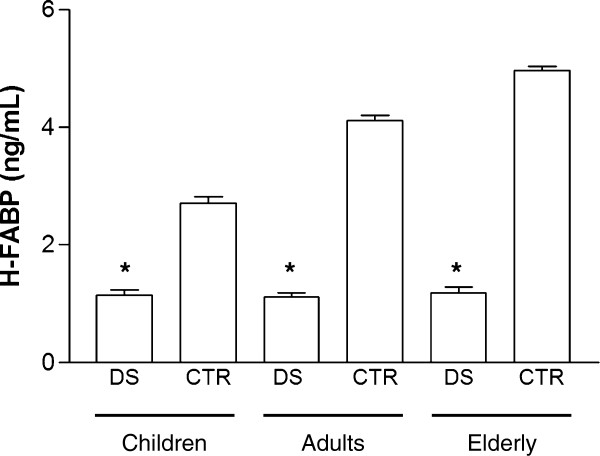
**H-FABP level in down syndrome people (DS) and healthy subjects (CTR) classified according to age.** DS and in CTR were subdivided in three groups according to age: the first included children (age 2–14 years); the second adults (age 20–50 years); the third elderly ( > 60 years). H-FABP was measured in each group and comparison has been performed both between DS and age-matched CTR and between the three DS and CTR age groups. *p < 0.001 *vs.* age-matched CTR, °p < 0.01 *vs.* adults CTR, °° p < 0.001 *vs.* children CTR.

Figure 2 shows that H-FABP level is statistically significantly lower in DS than in age-matched healty subjects (DS children 1.14 ± 0.41 ng/ml *vs.* healthy children 2.71 ± 0.51 ng/ml, p < 0.001; DS adults 1.11 ± 0.29 ng/ml *vs.* healthy adults 4.11 ± 0.35 ng/ml, p < 0.001; DS elderly 1.18 ± 0.23 ng/ml *vs.* healthy elderly 5.1 ± 0.15 ng/ml, p < 0.001).

Healthy subjects displayed a trend of increase in H-FABP level with aging (children *vs.* adults p < 0.001; elderly *vs*. adults p < 0.01). On the contrary, no difference has been observed in H-FABP level between children, adults and elderly DS (Figure 2).

## Discussion

The main finding of our study is that aging is normally associated with increased H-FABP level but this is not true in DS which display not only the same level in the different ages of life but have also lower level compared to their age-matched healthy subjects. Our study is in agreement with previous observation which described a striking increase in H-FABP value with aging [9] and, to our knowledge, is also the first comparing circulating H-FABP level in DS and healthy subjects.

The unchanged H-FABP level during aging in DS and the lack of atherosclerosis in these subjects prompted us to consider H-FABP as one potential molecule linking aging and atherosclerosis.

It is well know that aging is associated with reduced cellular proliferative potential, increased propensity to undergo death, elevated DNA damage and, at vascular level, increased expression of pro-inflammatory and leukocyte adhesion molecules and increased uptake of plasma lipoproteins, all key events that ultimately promote atherosclerosis [2]. Although the same mechanisms are present in DS, which also display an accelerated aging process compared to healthy subjects, anyway they resulted protected against atherosclerosis [10,11]. The mechanisms that may be involved in such protective effect are not well known. Anyway, our observation highlighted that reduced H-FABP level may contribute to this general protection.

Our study have some limitations, including the reduced sample size, the lack of correlation with bioclinical parameters and atherosclerosis-related risk factors. For these reasons, our data may be considered preliminary observations which need further investigation to better understand the relationship between H-FABP, atherosclerosis and coronary artery disease.

In conclusion, reduced H-FABP level during aging in DS may play a protective role against atherosclerosis. This observation highlighted a potential involvement of H-FABP in the relationship between aging and atherosclerosis.

## Consent

Written informed consent was obtained from the patient for publication of this report and any accompanying images.

## Abbreviations

DS: Down syndrome people; H-FABP: Heart-type fatty acid binding protein.

## Competing interests

The authors declare that they have no competing interests.

## Authors’ contributions

EV carried out the protein array procedure and wrote the manuscript. GD participated to the preparation of materials used in the experiment and participated to the experiment described in the text. ED contributed to the analysis and interpretation of data and revised it for intellectual content. MMCR senior author, conceived of the study, participated in its design and coordination. This work was supported by Banca Popolare di Milano Fundation Grant and by the University of Milan. All authors approved the final manuscript.

## References

[B1] More KJ Tabas I Macrophages in the pathogenesis of atherosclerosis Cell 2011 145 341 355 10.1016/j.cell.2011.04.005 21529710 PMC3111065

[B2] Wang JC Bennet M Aging and atherosclerosis: mechanisms, functional consequences, and potential therapeutics for cellular senescence Circ Res 2012 111 245 259 10.1161/CIRCRESAHA.111.261388 22773427

[B3] Fournier NC Richard MA Role of fatty acid-binding protein in cardiac fatty acid oxidation Mol Cell Biochem 1990 98 149 159 2266957 10.1007/BF00231379

[B4] Nagahara D Nagahara T Hashimoto A Takahashi T Kyuma M Hase M Tsuchihashi K Shimamoto K Early positive biomarker in relation to myocardial necrosis and impaired fatty acid metabolism in patients presenting with acute chest pain at an emergency room Cir J 2006 70 419 425 10.1253/circj.70.419 16565558

[B5] Basak K Ozbek M Bozkurt NC Ginis Z Gungunes A Unsal IO Cakal E Delibasi T Heart-type fatty acid binding protein (H-FABP): relationship with arterial intima-media thickness and role as diagnostic marker for atherosclerosis in patients with impaired glucose metabolism Cardiovasc Diabetol 2011 10 37 10.1186/1475-2840-10-37 21535886 PMC3112391

[B6] Murdoch JC Rodger JC Rao SS Down’s syndrome: an atheroma free model ? Br Med J 1977 2 226 228 10.1136/bmj.2.6081.226 141966 PMC1631400

[B7] Patterson D Molecular genetic analysis of down syndrome Human Genetic 2009 126 195 214 10.1007/s00439-009-0696-8 19526251

[B8] Ylä-Herttuala S Luoma J Nikkari T Kivimaki T Down’s syndrome and atherosclerosis Atherosclerosis 1989 76 269 272 10.1016/0021-9150(89)90110-X 2525042

[B9] Niizeki T Takeishi Y Takabatake N Shibata Y Konta T Kato T Kawata S Kubota I Circulating levels of heart-type fatty acid-binding protein in a general Japanese population: effects of age, gender, and physiologic characteristics Circ J 2007 71 1452 1457 10.1253/circj.71.1452 17721027

[B10] Dogliotti G Galliera E Licastro F Corsi MM Age-related changes in plasma levels of BDNF in Down syndrome patients Immun Ageing 2010 25 7 10.1186/1742-4933-7-2 PMC2841579 20181009

[B11] Corsi MM Dogliotti G Pedroni F Galliera E Malavazos AE Villa R Chiappelli M Licastro F Adipocytokines in Down’s syndrome, an atheroma-free model: Role of adiponectin Arch Gerontol Geriatr 2009 48 106 109 10.1016/j.archger.2007.10.011 18207581

